# Changing Shapes of Glycogen–Autophagy Nexus in Neurons: Perspective from a Rare Epilepsy

**DOI:** 10.3389/fneur.2015.00014

**Published:** 2015-02-04

**Authors:** Pankaj Kumar Singh, Sweta Singh

**Affiliations:** ^1^Department of Translational Medicine and Neurogenetics, Institut de Génétique et de Biologie Moléculaire et Cellulare (IGBMC), Illkirch, France

**Keywords:** autophagy, glycogen, polyglucosan, neurodegeneration, Lafora disease

## Abstract

In brain, glycogen metabolism is predominantly restricted to astrocytes but it also indirectly supports neuronal functions. Increased accumulation of glycogen in neurons is mysteriously pathogenic triggering neurodegeneration as seen in “Lafora disease” (LD) and in other transgenic animal models of neuronal glycogen accumulation. LD is a fatal neurodegenerative disorder with excessive glycogen inclusions in neurons. Autophagy, a pathway for bulk degradation of obsolete cellular constituents also degrades metabolites like lipid and glycogen. Recently, defects in this pathway emerged as a plausible reason for glycogen accumulation in neurons in LD, although some contradictions prevail. Albeit surprising, a reciprocal regulation of autophagy by glycogen in neurons has also just been proposed. Notably, increasing evidences of interaction between proteins of autophagy and glycogen metabolism from diverse model systems indicate a conserved, dynamic, and regulatory cross-talk between these two pathways. Concerning these findings, we herein provide certain models for the molecular basis of this cross-talk and discuss its potential implication in the pathophysiology of LD.

## Introduction

A common feature of many neurodegenerative disorders is the defect in protein quality control mechanisms including ubiquitin proteasome system (UPS) and macroautophagy (hereafter referred as autophagy) leading to biogenesis and accumulation of protein aggregates or inclusions (1). During the last decade, autophagy has been increasingly recognized as the primary reason behind pathogenesis of several neurodegenerative disorders. Defects in autophagy perturb neuronal functions, progressively leading to neurodegeneration (2). Beyond proteolysis, autophagy also plays a pivotal role in nutrient recycling and metabolic homeostasis by degrading lipids and glycogen (3). Surprisingly, while proteolytic dysfunction of autophagy is well recognized to underlie pathogenesis of neurodegenerative disorder, influence of its metabolic aspect is comparatively less explored.

In animals, glycogen has evolved as an efficient means of energy storage. In addition, the metabolism of this carbohydrate in the liver helps to maintain the blood and cerebral glucose level within the physiological limits during hypoglycemia and starvation. Intriguingly, despite glucose being the preferential energy source for the neurons and the presence of molecular machinery to synthesize glycogen, neurons synthesize very low glycogen under physiological conditions compared to most other cell types in animals (4). Glycogen or glycogen like inclusions called polyglucosan bodies, nevertheless do accumulate in neurons in (a) pathologies like Pompe disease (5), Lafora disease (LD) (6), Alzheimer’s disease (AD) (7) amyotrophic lateral sclerosis (8), adult polyglucosan body disease (APBD) (9), (b) under pathophysiological conditions like diabetes (10), hypoxia (11), and during aging (9). Surprisingly, this accumulation correlates with neurodegeneration in Pompe disease (5), in LD (6), in fly/mouse models expressing constitutive active glycogen synthase (GS) in neurons (12), and with reduced neuronal functions during aging (13). These findings, therefore, suggest that glycogen or related inclusion bodies are a pathogenic entity in the brain.

Remarkably, a direct impact of glycogen or its metabolic/regulatory proteins in control of neuronal functions is slowly growing. Thus, while induced glycogen accumulation in neurons promotes neurodegeneration, the prevention of polyglucosan accumulation in neurons by knocking down GS improves neurological functions and increases life span in aged fly (13). Notably, accumulation of carbohydrate inclusion (9) and reduced autophagy activity (14) in neurons, are two independent hypotheses proposed for the decline of neuronal functions with aging. Autophagy degrades glycogen and a recent indication of converse regulation of autophagy by glycogen or its constituent proteins, has suggested a direct functional link between glycogen and this proteolytic pathway. Mounting evidences for interaction between proteins of these two pathways has further compelled us to uncover the molecular mechanism linking autophagy activity, glycogen accumulation, and neuronal survival. This review summarizes all the recent findings of evolving autophagy–glycogen connection and its contribution to pathogenesis of neurodegenerative disorders, particularly LD.

## Neuronal Glycogen Metabolism: Growing Understanding from a Rare Epilepsy – “Lafora Disease”

During the last decade, understanding the pathophysiological mechanism of a rare progressive myoclonus epilepsy “LD” has uncovered many interesting aspects of neuronal glycogen metabolism. LD is a fatal neurodegenerative disorder characterized by accumulation of insoluble, hyperphosphorylated, and less branched form of glycogen called Lafora bodies (LBs or polyglucosan more commonly) in several tissues including neurons of patients and mouse models (6, 15–17). Laforin and malin, two proteins pathologically linked to LD, were found to promote neuronal survival by restricting glycogen synthesis (18). A complex of these proteins was reported to keep neuronal glycogen synthetic machinery constitutively silent by enforcing GS inactivation and degradation of protein targeting to glycogen (PTG, an adaptor subunit of protein phosphatase 1 and activator of glycogen synthesis), through proteasome (18). This report is, however, challenged by subsequent findings demonstrating no alteration in the level of PTG (19, 20) and conflicting reports about GS activity in the brain of LD mouse models (17, 19, 20). Increased phosphorylation of glycogen in the absence of glucan phosphatase laforin is hypothesized as another reason behind neuronal glycogen accumulation (19). This hypothesis has also been recently challenged by Gayarre et al. (21), who show that the phosphatase activity of laforin is dispensable for prevention of LB formation and rescuing LD pathogenesis. An inadequate understanding of the precise regulatory role of LD proteins in glycogen metabolism therefore makes it inconceivable, how polyglucosan biogenesis is elicited in LD.

Inhibition of glycogen synthesis prevents polyglucosan accumulation and neurodegeneration in double knockout mice of PTG/laforin (22), GS/laforin (23), and PTG/malin (24). Thus, it appears that glycogen is regulated primarily at the level of its synthesis in neurons. Interestingly, potential significance of glycogen degradation in buildup of neuronal glycogen is also now gaining considerable attention. Thus, subsiding a previous report about absence of glycogen and its degradative enzyme glycogen phosphorylase in neurons, Saez et al. have demonstrated the presence of glycogen and its rapid turnover by glycogen phosphorylase (GP) (4). Furthermore, identification of autophagy defect in LD particularly in the presence of functional glycogen phosphorylase suggests this pathway as an alternative route of neuronal glycogen degradation (25–27). Although the relative contribution of glycogen phosphorylase and autophagy in glycogen degradation is undetermined, autophagy axis is steadily gaining more attention.

## Involvement of Autophagy in Neuronal Glycogen Degradation: Current Understanding and Emerging Controversies

Several indirect evidences now exist that as in many other tissues, autophagy may degrade the glycogen in neurons. For example, defects in lysosomal enzyme “acid alpha-glucosidase (GAA)” are linked to glycogen accumulation in many tissues including neurons (5). Recently, autophagy defects at the level of compromised autophagosome formation, defective lysosomal structure, activity, and endosomal–lysosomal trafficking have been found in the brain of LD mice models (25–27). Further, as a rather direct evidence, Gayarre et al. have reported that forestalling autophagy defects in EPM2A^−/−^ mice correlates with the absence of LB in the brain (21). These authors by expressing transgene encoding either active laforin (wild type, LAFWT) or its phosphatase inactive mutant (LAFC265S) in laforin-deficient mouse (Epm2a^−/−^) observed that both proteins are equally efficient in forestalling autophagy defects and prevent LBs formation in Epm2a^−/−^ mouse. Still at molecular level, a direct role of laforin in both these processes and involvement of autophagy in neuronal glycogen degradation is not clear from this study. Nonetheless, together these findings convincingly indicate possible involvement of autophagy in neuronal glycogen degradation.

In contrast to this, Kakhlon et al. (28) have demonstrated failure of autophagy to degrade polyglucosan in a primary neuronal model of polyglucosan accumulation created by knockdown of glycogen branching enzyme (Gbe1). These authors failed to detect polyglucosan within any of the vesicular compartments of autophagy even after inducing autophagy by rapamycin (mammalian target of rapamycin, mTOR, inhibitor) treatment and starvation. Moreover, they noticed that rapamycin decreases polyglucosan accumulation by inhibiting GS activity and that this decrease is not prevented by autophagy blockade.

In the middle of this controversy, a completely new prospect of autophagy–glycogen relation emerged when Duran et al. (29) showed that in brain, autophagy activity corresponds to cellular glycogen level. The authors using malin-deficient mice (malin^KO^, an LD model) and double knockout mice with either partial (malin^KO^ + MGS^Het^) or complete (malin^KO^ + MGS^KO^) disability to store glycogen owing to partial (MGS^Het^) or complete (MGS^KO^) lack of GS in the brain showed that, reduction in glycogen level significantly rescued autophagy impairments and neurodegeneration. Conversely, they found that transgenic mice overexpressing, PTG (PTG^OE^) or non-inactivatable form of GS 9A-MGS (9A-MGS^OE^) show increased glycogen accumulation and autophagy impairments in the brain. Based on these observations, the authors claim to have resolved the question whether glycogen accumulation is a cause or consequence of autophagy defect, favoring the former possibility. This could be possible that in neurons, polyglucosan buildup hamper vesicular trafficking important for proper autophagy function. The enigma will, however, continue until it is ascertained that altered activity/level of GS/PTG protein does not modulate autophagy function intrinsically and has no direct involvement in this process. The reason for this concern is made apparent in the following sections.

Although autophagy-mediated glycogen degradation is well established, evidences for its existence in neurons are so far still in the preliminary stage. In this regard, conflicting reports like autophagy does not degrade neuronal polyglucosan (28), but at the same time, autophagy might prevent LBs accumulation (21), has created confusion as to whether, and under what context, autophagy could degrade neuronal glycogen. The discrepancy is further compounded by the finding that glycogen level determines autophagy activity (29). We believe that all these findings could have distinct physiological reasons and in a larger context, determining mutual regulation of autophagy and glycogen metabolism could help us to understand these discrepancies. Therefore, an in-depth discussion of these findings is prerequisite to evoke future research interest in the field to suggest the possible directions of investigation.

Herein, going against our own belief, we have first discussed how autophagy might fail to degrade neuronal glycogen. Afterwards, we have provided logical assumptions to contradict models rejecting the role of autophagy in neuronal glycogen degradation. Finally, envisaging certain models showing how proteins of these two cellular pathways may interact and regulate each other, we support the role of autophagy in neuronal glycogen degradation and insist a fresh revisit to investigate this metabolic nexus.

## Possible Prospects of Polyglucosan Degradation by Autophagy

Glycogen is seemingly pathogenic to neurons, yet it is poorly understood why or how the neuronal machinery fails to clear it. It is noteworthy that neurons preferentially synthesize polyglucosan over glycogen. The term “Polyglucosan or Polyglucosan bodies” is attributed to structure, which represents less branched form of glycogen. These are hyperphosphorylated, poorly branched, amylase insensitive, and insoluble entities in contrast to glycogen (19). Perhaps, due to these structural and biochemical differences, the protein/s recruiting glycogen for autophagic degradation might fail to recognize polyglucosan. This opinion is based on finding that LD protein laforin shows more affinity for polyglucosan than glycogen (30). Therefore, altered affinity of proteins indispensable for glycogen degradation toward polyglucosan would obviously affect its metabolism. An interesting quest to this end would thus be to find out proteins mediating glycogen–autophagy interaction and identify their affinity for polyglucosan.

Glycogen degradation in tissues like liver and heart particularly under starvation or hypoglycemic conditions primarily serves the purpose of instant energy supply by providing glucose. The neuronal energy demand under such conditions is, however, compensated by surrounding glial cells (31) and therefore even under glycogenolytic signals, polyglucosan degradation in neurons will prove futile with no metabolic/physiological advantage to them. The basal autophagy operates at low level in neurons and it is quite resilient to induction under glycogenolytic condition like starvation that otherwise induces autophagy in the liver and heart tissues (31). Thus, with limited autophagy induction, neurons seem more prone to accumulate polyglucosan inclusions when the rate of synthesis of these inclusions exceeds the degradative capacity of autophagy. An interesting exploration would thus be to see if compensatory autophagy induction clears extra-neuronal polyglucosan. This will also uncover whether there exists any fundamental difference in metabolisms of this entity between neuronal and non-neuronal tissues.

These hypotheses about failure of autophagy to degrade polygucosan are, however, unjustified in realms of reports that, a structurally similar storage carbohydrate of plant; “starch” is degraded by autophagy (32) and autophagy might inhibits polyglucosan accumulation (21). How then Kakhlon et al. might have failed to observe autophagic clearance of polyglucosan? Autophagy is a selective process and specific adaptor proteins determine its substrate specificity in signal-dependent manner (33). Therefore, one reason could be that rapamycin treatment and starvation are not specific signals for glycogen degradation through autophagy in neurons. In fact, the extent of *in vivo* induction of autophagy in brain by both these manipulations is quite debatable. Interestingly, as discussed in the following section, these signals might rather inhibit glycogen recruitment to autophagosome. Thus, specific pharmacological or genetic manipulations that induce autophagy in brain must be tested before abandoning autophagy’s role in neuronal polyglucosan clearance. Additionally, it would be interesting to investigate whether such manipulations also manage to clear extra-neuronal polyglucosan.

With emergence of glycogenic proteins at different steps of autophagy (recruitment, and autophagosome synthesis), as discussed in the following section, glycogen metabolism seems a relatively more complex process than believed to be previously. Thus, it might be possible that despite active autophagy, loss of a specific glycogenic protein hampers glycogen degradation by perturbing its recruitment to autophagosome. Exploration of the role of glycogen branching enzyme (Gbe1) in this aspect would not only assert this hypothesis but would also settle the controversy stirred by Kakhlon et al. study.

Another intriguing exploration would be the impact of phosphorylation–dephosphosphorylation of glucosyl unit of glycogen over its autophagic degradation. Polyglucosan is hyperphosphorylated compared to glycogen and this biochemical modification of glucosyl unit in starch assists its degradation (34). Understanding the effect of this modification in autophagic degradation of starch might therefore uncover whether and how polyglucosan could be degraded by autophagy machinery in animal. Together, autophagy-mediated polyglucosan degradation in neurons is possibly provided, the process of autophagy induction, polyglucosan recruitment to autophagosome and lysosomal activity are intact, and the burden of polyglucosan remains within the physiological limit of autophagy capacity.

## Autophagy–Glycogen Cross-Talk – A New Paradigm

The metabolic fate of glycogen (synthesis vs. degradation) is a function of active communication among its constituent proteins, which are perhaps also involved in other cellular processes. For example, GS has recently been found to interact with Atg8 (35), its human ortholog GABARAPL1 (36), and to regulate autophagy. Moreover, proteins like laforin, malin are supposed to play a role in autophagosome synthesis (25, 26). In addition, there are proteins like starch-binding domain-containing protein 1 (Stbd1) (37) and receptor of activated protein kinase C 1 (Rack1) (38), with poorly defined functions but, both interact with players of glycogen metabolism and autophagy (Table 1). The studies performed under loss or gain of function of these proteins, therefore would obviously tell the eventual metabolic fate of glycogen resulting from number of molecular changes happening “within, around, and outside the domain of glycogen granules” and consequent cellular adaptation. The autophagy–glycogen relationship derived from such studies therefore, should be analyzed with extreme caution.

**Table 1:** **The so far identified potential proteins with dual roles in glycogen metabolism and autophagy**.

Proteins	Co-localization with glycogen/autophagosome	Loss of function phenotype		Model system	Reference
	
		Glycogen level	Autophagy activity	
Laforin	+/Not yet known	↑	↓	Mouse	(19, 25)
Malin	+/Not yet known	↑	↓	Mouse	(26, 45)
Stbd1	+/+	Not yet known	Not yet known	HepG2 hepatoma cells	(37)
Rack1	+/+	↓	↓	Fly	(38)
GS (muscle)	+/+	↓	Not yet known	Fly/mouse	(35, 46)

**Figure 1:**
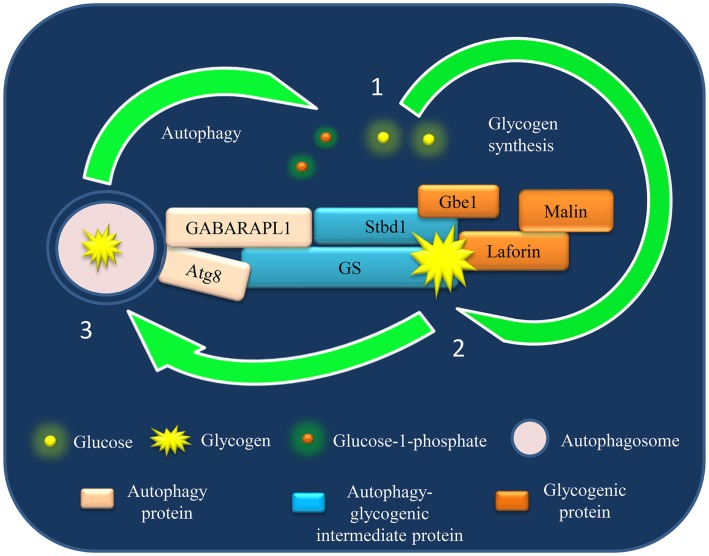
**A model of autophagy–glycogen proteins interaction based on current understanding and its potential implication in Lafora disease (LD)**. The schematic representation shows the identified interaction until now between the proteins of glycogen metabolism and autophagy network. The physiological implication of these interactions in context of Lafora disease (LD) is discussed in this review.

*The dual nature is assigned if protein already known to function in one pathway co-localizes, interacts (as shown in Figure 1) with other pathway components, or even if its loss affects both the pathways*.

With unfolding of interaction between protein of glycogen metabolism and autophagy, it seems that these two pathways are in continuous cross-talk and depending upon specific metabolic signals and/or, active/inactive state of specific proteins, they regulate each other in a manner beneficial to the cell. For instance, GS–Atg8 interaction depends upon GS activity as mutation affecting GS ability to bind with its allosteric activator glucose-6-phospate not only inhibits its activity but also perturbs its interaction with Atg8 (35). Conversely, GS insensitive for starvation-induced activity suppression by GS kinase 3 beta (GSK3β), still interact with Atg8 during starvation (35). Therefore, a dynamic balance between the relative ratio of free GS (for glycogen synthesis) and GS–Atg8 (determining autophagy activity) is essential for both these process to take place under basal condition. An activity-dependent change in GS–Atg8 interaction therefore would perturb the cellular availability of free Atg8 for autophagosome formation and autophagy activity as such, or recruitment of a specific substrate like glycogen to the autophagosome. Going by this hypothesis, reduced GS–Atg8 interaction and perturbed recruitment of polyglucosan in autophagosome upon GS inhibition by rapamycin/starvation could be a reason behind Kakhlon et al. failure to see polyglucosan in autophagic compartment despite autophagy induction.

## Relevance of Glycogen–Autophagy Cross-Talk in LD

In neurons, change in the sub-cellular localization of GS and not just its increased level or activity determines glycogen synthesis (18). Therefore, signals that induce movement of GS from nucleus to the cytoplasm particularly in the brain might promote glycogen synthesis with concomitant suppression of autophagosome synthesis due to increased GS–Atg8 interaction. Laforin and GS both show nuclear–cytoplasmic translocation in response to glycogen availability (39, 40) and laforin interacts with GS (41). Therefore, loss of laforin-induced cytoplasmic translocation of GS triggering glycogen synthesis and perturbing autophagy could be a plausible reason of LB biogenesis. This functional model of GS–Atg8 also explains the molecular basis of autophagy defect seen in malin knockout mice (malin^KO^) with increased GS level (29) and unaltered mammalian target of rapamycin activity (26). This also unequivocally explains how the loss of GS in malin-GS double knockout mice (malin^KO^ + MGS^Het^ and malin^KO^ + MGS^KO)^ could rescue autophagy defect and prevent glycogen accumulation (29).

## Future Directions

The discussed GS–Atg8 interaction is just one example of many such possible interactions between the proteins of these two cellular pathways. As both these pathways encompass several proteins, this cross-talk could supposedly be more complex involving several axis, with each composed of a specific set of proteins functional in one or other physiological conditions. For example, both GS and Stbd1 bind with glycogen and contain Atg8-interacting motif (AIM) predicted to be required for selective degradation of cargo (perhaps glycogen). Moreover, Stbd1 interacts with GS and other glycogenic proteins like laforin and debranching enzyme (42). In the light of such a complex interaction network (Figure 1), it would be difficult to predict the mechanism of Stbd1- or GS-mediated glycogen degradation by autophagy at this stage. Nevertheless, it can be speculated that, perhaps several independent and redundant mechanisms operate for dynamic regulation of autophagy–glycogen axis to meet metabolic demands under different physiological conditions.

Importantly, since these interactions are identified in cells of diverse origins, this cross-talk appears to be a conserved mechanism. This though could have cell/tissues specificity in context of the proteins involved particularly considering the tissue-specific isoforms of several glycogen metabolism proteins (43) and the fact that, glycogen proteome itself shows differences in its constituent proteins among various tissues (44). Cumulatively, these evidences postulate the existence of an intricate functional relationship between glycogen metabolism and autophagy and are intriguingly encouraging to explore the autophagy–glycogen communication with a fresher perspective, particularly in neurons. For this, animal models of glycogen storage (GS, PTG overexpression mice) and deficiency (GS knockout mice) can be utilized to study autophagy activity in brain. Likewise, autophagy-related gene (Atg)-deficient mice could be beneficial in investigating the status of neuronal glycogen metabolism.

## Conclusion

The hypotheses proposed herein based on current evidences fully support autophagy-mediated glycogen degradation in neurons. In this direction, therefore, we urge to identify additional components of glycogen metabolism and autophagy machinery that interact and mutually regulate each other perhaps, under varied physiological conditions. Furthermore, in order to identify a direct link between these two pathways, an immediate challenge is to generate an appropriate cellular/animal model for dynamic monitoring of both these pathways simultaneously. As autophagy defect is seen in a number of neurodegenerative disorders and glycogen accumulation induces neurodegeneration, understanding a causal relationship between the two should be one of the prime focuses of future investigations in order to enhance the therapeutic potential of the diseases like LD, and to target proteins of glycogen metabolism as novel therapeutic interventions.

## Author Contributions

PS; conception and design. PS, SS; writing and discussion.

## Conflict of Interest Statement

The authors declare that the research was conducted in the absence of any commercial or financial relationships that could be construed as a potential conflict of interest.
